# A comprehensive genomic characterization of esophageal squamous cell carcinoma: from prognostic analysis to in vivo assay

**DOI:** 10.1186/s40880-016-0142-y

**Published:** 2016-08-11

**Authors:** Yuan-Bin Chen, Wei-Hua Jia

**Affiliations:** Sun Yat-sen University Cancer Center, State Key Laboratory of Oncology in South China, Collaborative Innovation Center for Cancer Medicine, Guangzhou, 510060 Guangdong P. R. China

**Keywords:** Esophageal squamous cell carcinoma, Copy number variation, Prognostic analysis

## Abstract

**Background:**

Esophageal squamous cell carcinoma (ESCC) is a leading cause of cancer death worldwide and is characterized by numerous genetic mutations. TNM staging is not sufficient for predicting patient outcomes. Additionally, ESCC shows poor responsiveness to chemotherapy and radiation. Thus, there is an urgent need to find efficient therapy targets. Previous ESCC high-throughput genomic studies have lacked intensive survival analysis, particularly for copy number variation (CNV) and the genes involved.

**Main body:**

In the study “Genomic Characterization of Esophageal Squamous Cell Carcinoma Reveals Critical Genes Underlying Tumorigenesis and Poor Prognosis” recently published in the *American Journal of Human Genetics*, we comprehensively analyzed the effects of CNVs, mutations, and relative gene expression on patient outcomes. To validate our findings for our 67 sequencing samples, we collected a 321-patient retrospective cohort with detailed 5-year follow-up information and carried out univariate and multivariate survival analyses. In addition, the biological functions of the survival predictors in ESCC were investigated both in vitro and in vivo.

**Conclusions:**

We found the independent ESCC survival predictors and potential therapy targets. Nevertheless, the effects of numerous low-frequency mutations need to be explored using larger sample sequencing. Overall, constructing multi-gene prognostic signatures will remain a great challenge in the future.

## Background

Esophageal cancer is the eighth most common cancer and the sixth leading cause of cancer-related death in the world, with approximately 456,000 new cases per year globally [1–4]. There are two main esophageal cancer histological subtypes: esophageal squamous cell carcinoma (ESCC) and esophageal adenocarcinoma [5]. ESCC accounts for 80% of esophageal cancer cases worldwide, and the risk is influenced by environmental factors (alcohol consumption and tobacco use) and genetic factors. Only 15%–25% ESCC patients survive for 5 years [6, 7].

Although staging of the tumor is important for determining the therapeutic strategy, it cannot precisely predict prognosis. Thus, a signature for classifying patients as potential therapy responders or non-responders would be of great clinical use if performed using initial diagnostic biopsies.

The poor responsiveness of ESCC to chemotherapy and radiation is a main reason for poor patient outcomes. Moreover, efficient targeted medicine for ESCC treatment is lacking to date. Therefore, the mechanisms underlining the poor prognosis of this disease are essential for finding therapy targets.

Several ESCC high-throughput genomic sequencing studies have been reported recently [8–11]; however, none has comprehensively evaluated both the effects of copy number variations (CNVs) and mutations on patient prognosis.

## Main body

In our recent article entitled “Genomic Characterization of Esophageal Squamous Cell Carcinoma Reveals Critical Genes Underlying Tumorigenesis and Poor Prognosis” published in the *American Journal of Human Genetics*, we reported a high-throughput genomic sequencing study of ESCC. By detecting and characterizing somatic variants, we comprehensively analyzed the effects of CNVs, mutations, and relative gene expression on patients’ overall survival (OS). For essential genes, further biological experiments were carried out in vitro and in vivo. The goal was to identify critical mutations underlying the poor prognosis of ESCC and to find potential prognosis makers and therapy targets [12].

In the 67 samples comprising the sequencing cohort, 19,434 mutations were found in exon regions of the ESCC genome. Using MutSigCV software [13], tumor protein p53 (*TP53*), cyclin-dependent kinase inhibitor 2A (*CDKN2A*), notch homolog 1, translocation-associated (Drosophila) (*NOTCH1*), and nuclear factor, erythroid 2 like 2 (*NFE2L2*) were identified as significantly mutated genes. These four genes were then subjected to OS analysis. The results showed that patients harboring *NOTCH1* mutations had a longer lifespan after surgery than those without mutations. In addition, using a cohort of independent 321 samples, we found that individuals with lower *NOTCH1* expression had a higher 5-year OS rate than those with higher *NOTCH1* levels. Multivariate Cox regression analysis indicated that after adjustment for age, sex, tumor stage, smoking, and alcohol consumption, *NOTCH1* expression was significantly associated with OS. These findings suggest that *NOTCH1* might play an essential role in ESCC progression.

Compared with mutations, we found much higher frequencies of CNVs in ESCC. To explore their influence on survival, both CNVs and expression of the associated genes were detected by quantitative polymerase chain reaction (qPCR). A number of CNVs and genes were found to be associated with poor patient outcomes.

MYB proto-oncogene like 2 (*MYBL2*), a reported cell cycle regulator, showed elevated gene copy numbers in 70% of the tumors subjected to whole-genome sequencing. Its transcripts and protein were also overexpressed in ESCC compared with adjacent normal tissues. Both the gene copy number and expression of *MYBL2* showed negative effects on individuals’ survival. In vitro studies demonstrated that overexpression of *MYBL2* increased proliferation in ESCC cell lines.

Non-coding RNA plays an important role in tumorigenesis and development. However, previous comparative genomic hybridization studies mainly focused on coding genes. In our study, a CNV-harbored microRNA, *miR*-*4707*-*5p*, was found to be significantly overexpressed in tumors, and individuals with high *miR*-*4707*-*5p* levels exhibited worse prognosis than those with low *miR*-*4707*-*5p* levels. An in vitro pilot experiment revealed that *miR*-*4707*-*5p* has a strong ability to promote cell migration and invasion. The pro-metastasis ability of *miR*-*4707*-*5p* was confirmed in two different mouse tumor metastasis models. Furthermore, through mechanism studies, we found that *miR*-*4707*-*5p* can decrease E-cadherin by targeting adenosine deaminase, RNA specific B1 (*ADARB1*), in turn promoting cell metastasis. Therefore, the CNV-*miR*-*4707*-*5p*-*ADARB1*-E-cadherin axis might be a target of ESCC therapy.

Interestingly, VANGL planar cell polarity protein 1 (*VANGL1*), a novel high-frequency mutant gene that we found, showed no association with ESCC prognosis. Thus, *VANGL1* mutation might play a role only at early stages of the neoplastic process.

Although a list of prognosis predictors was found, we could not rule out the possibility that low-frequency mutations also have an impact on prognosis, at least for particular individuals. However, due to the limited sample size, the association between these low-frequency mutant genes and ESCC prognosis could not be analyzed in this study. As cancer occurrence and development are the result of the participation of multiple genes, a multi-gene prognostic signature would be more convincing, though this is difficult to realize because of the limited sample size, low frequency of mutations, and complexity of genomic variants.

## Conclusions

Through a comprehensive genomic study and gene expression analysis, we identified a number of independent prognosis predictors. We showed that at least two of them, *MYBL2* and *miR*-*4707*-*5p*, are involved in ESCC cell malignant transformation and might be a basis for poor ESCC prognosis. Considering the decreasing cost of sequencing, experiments with larger sample sizes and more serviceable algorithms would help in the future search for a multi-gene prognostic signature.
